# Femtosecond Laser Irradiation to Zirconia Prior to Calcium Phosphate Coating Enhances Osteointegration of Zirconia in Rabbits

**DOI:** 10.3390/jfb15020042

**Published:** 2024-02-11

**Authors:** Hirotaka Mutsuzaki, Hidehiko Yashiro, Masayuki Kakehata, Ayako Oyane, Atsuo Ito

**Affiliations:** 1 Center for Medical Science, Ibaraki Prefectural University of Health Sciences, 4669-2 Ami, Ibaraki 300-0394, Japan; 2 Department of Orthopaedic Surgery, Ibaraki Prefectural University of Health Sciences Hospital, 4773 Ami, Ibaraki 300-0331, Japan; 3 Research Institute for Advanced Electronics and Photonics, National Institute of Advanced Industrial Science and Technology (AIST), AIST Tsukuba Central 2, 1-1-1 Umezono, Tsukuba, Ibaraki 305-8568, Japan; hidehiko.yashiro@aist.go.jp (H.Y.); kakehata-masayuki@aist.go.jp (M.K.); 4 Nanomaterials Research Institute, National Institute of Advanced Industrial Science and Technology (AIST), AIST Tsukuba Central 5, 1-1-1 Higashi, Tsukuba, Ibaraki 305-8565, Japan; a-oyane@aist.go.jp; 5 Health and Medical Research Institute, National Institute of Advanced Industrial Science and Technology (AIST), AIST Tsukuba Central 6, 1-1-1 Higashi, Tsukuba, Ibaraki 305-8566, Japan; atsuo-ito@aist.go.jp

**Keywords:** zirconia, calcium phosphate, femtosecond laser, bone formation, bone-bonding ability

## Abstract

Calcium phosphate (CaP) coating of zirconia and zirconia-based implants is challenging, due to their chemical instability and susceptibility to thermal and mechanical impacts. A 3 mol% yttrium-stabilized tetragonal zirconia polycrystal was subjected to femtosecond laser (FsL) irradiation to form micro- and submicron surface architectures, prior to CaP coating using pulsed laser deposition (PLD) and low-temperature solution processing. Untreated zirconia, CaP-coated zirconia, and FsL-irradiated and CaP-coated zirconia were implanted in proximal tibial metaphyses of male Japanese white rabbits for four weeks. Radiographical analysis, push-out test, alizarin red staining, and histomorphometric analysis demonstrated a much improved bone-bonding ability of FsL-irradiated and CaP-coated zirconia over CaP-coated zirconia without FsL irradiation and untreated zirconia. The failure strength of the FsL-irradiated and CaP-coated zirconia in the push−out test was 6.2–13.1-times higher than that of the CaP-coated zirconia without FsL irradiation and untreated zirconia. Moreover, the adhesion strength between the bone and FsL-irradiated and CaP-coated zirconia was as high as that inducing host bone fracture in the push-out tests. The increased bone-bonding ability was attributed to the micro-/submicron surface architectures that enhanced osteoblastic differentiation and mechanical interlocking, leading to improved osteointegration. FsL irradiation followed by CaP coating could be useful for improving the osteointegration of cement-less zirconia-based joints and zirconia dental implants.

## 1. Introduction

Ceramic implants, such as zirconia and alumina-zirconia composites, are advantageous over metallic implants, owing to their excellent chemical stability, biocompatibility, high wear resistance, and artifact-free magnetic resonance imaging (MRI) [1,2,3,4,5,6]. Meanwhile, metallic implants have the risk of inducing metal hypersensitivity [7], metallosis [8], and pseudotumors [9], although they show acceptable biocompatibility and favorable mechanical properties. Metallic implants also cause severe MRI artifacts [5,6], which interfere with diagnosis or making appropriate treatment decisions in postoperative evaluation of the implant and surrounding tissues.

However, zirconia and alumina-zirconia composite ceramic implants present a challenge for acquiring bone-bonding ability [10]. While bone-bonding ability is simply obtained on metallic implants by thermal spray coating of osteoconductive calcium phosphate (CaP), the high temperature over 1100 °C used to melt CaP deteriorates the mechanical properties of zirconia, due to crack formation and phase transformation. Thus, zirconia-based implants are used without CaP coatings. If necessary, bone cement is used in clinical practice, although such an approach may induce the development of bone cement implantation syndrome [1,11]. Coating zirconia with CaP using a process with low thermal effect is crucial for improving the bone-bonding ability of zirconia without deteriorating mechanical properties. Zirconia-based implants have previously been coated by CaP without severe heating of substrates using aqueous solution processing [12,13,14,15,16,17,18], aerosol deposition [19], magnetron sputtering [20], and ion-beam-assisted deposition [21]. Moreover, the bone-bonding ability of CaP-coated implants improved relative to uncoated ones in animal studies [12,16,22]. However, achieving a high adhesion strength between the CaP coating and zirconia-based implant remains a challenge. A low adhesion strength at the CaP–implant interface eventually causes a low adhesion strength between the bone and implant.

Recently, surface roughening of zirconia at 100-micrometer scale was carried out to improve CaP coating adhesion [23,24,25]. In this technique, a nano-second Nd:YAG laser was used to produce micro-textures on zirconia, and a CO_2_ laser was used to sinter a CaP coating (prepared by dip-coating) on the micro-textured zirconia.

In our previous study, a femtosecond laser (FsL) was used to produce tens-of-micron-sized craters that had linear submicron grooves on their concave surface on zirconia (3 mol% yttrium-stabilized tetragonal zirconia polycrystal: 3Y-TZP), thereby improving CaP coating adhesion [13]. Our previous CaP coating was carried out using a three-step procedure: (i) FsL irradiation on zirconia substrates; (ii) alternate dipping treatment; and (iii) immersion in a supersaturated CaP solution (hereafter, referred to as ‘solution process’) [13]. In the first step of surface roughening, FsL was employed, since FsL irradiation leads to much less thermal damage to the bulk properties of zirconia compared to nanosecond or longer laser pulses, despite the transient high temperature (over the melting point: 2115 °C) locally at the surface. This is because ultrashort laser pulses make the thermal transport distance negligibly short in contrast to that of nanosecond laser pulses [26,27]. In more detail, the strong electric field or high intensity of FsL pulses causes the nonlinear absorption or multi-photon absorption and ionizes the surface of the zirconia. The ionized materials are ejected from the surface through the mechanism known as Coulomb explosion, which is a nonthermal process. The time required for Coulomb explosion is shorter than that required for the thermal diffusion; therefore, most of the energy absorbed by the non-linear absorption is transported to the ablation products. Thus, the thermal effect of FsL pulses on the ablated surface is small compared with that of nanosecond or longer laser pulses [27]. Furthermore, the tens-of-micron-sized craters and submicron grooves on the zirconia enhance the osteogenic differentiation of mesenchymal stem cells [28]. In the second step, the FsL-irradiated zirconia was alternately dipped in calcium and phosphate solutions three times for the deposition of a CaP underlayer. In the third step, the thus-treated zirconia was immersed in a supersaturated CaP solution to increase the thickness of the CaP coating.

In our previous CaP coating technique describe above, the tens-of-micron- and submicron-scale surface roughening effectively increased CaP coating adhesion [13], without causing noticeable phase transformation in the zirconia from tetragonal to monoclinic [29]. However, the bone-bonding abilities of FsL-irradiated and CaP-coated zirconia substrates have yet to be clarified. In the present study, we refined our previous CaP coating technique using pulsed laser deposition (PLD) in place of the alternate dipping treatment (in the second step) and employing clinically available infusions and injection fluids as sources for the supersaturated CaP solution (in the third step). The PLD process can precisely control the composition and structure of CaP [30]. In addition, the PLD process can be used for CaP coating on complex-shaped substrates such as zirconia screws [31]. In the present study, the PLD process was carried out at the substrate temperature of 20–25 °C for the deposition of a CaP underlayer. Clinically available infusions and injection fluids were utilized to improve the biosafety of coated CaP for clinical applications of CaP-coated zirconia. The infusions and injections used were water, calcium-containing solutions (two types with different concentrations), phosphorus-containing solutions (two types with different concentrations), a NaHCO_3_ solution (an alkalizer) to increase pH, and saline to adjust ionic strength. CaP-coated zirconia with and without FsL irradiation (Groups A and B, respectively) along with untreated zirconia (Group C) were implanted in rabbit tibial metaphyses. This study aimed to clarify the effect of FsL irradiation on the bone-bonding ability of CaP-coated zirconia implants in rabbits. After implantation for four weeks, the push-out strength of the implant and bone-to-implant contact ratio were evaluated. We hypothesized that FsL irradiation would improve the bone-bonding ability of CaP-coated zirconia.

## 2. Materials and Methods

### 2.1. Preparation of Zirconia Implants

Quadratic prism implants of zirconia were prepared by compacting 3Y-TZP powders (TZ-3YB-E, Tosoh Co., Tokyo, Japan) using a cold isostatic pressing (CIP) technique followed by sintering at 1350 °C for 2 h and cutting. Four rectangular planes with dimensions of 2.4 mm (Y- and Z-directions) × 21 mm (X-direction) were subjected to wet-polishing to mirror quality surfaces (Ra < 0.05 µm). The implants were ultrasonically washed three times with acetone and then dried at room temperature (20–25 °C).

The implants were divided into Groups A, B, and C, where Group A implants were subjected to FsL irradiation followed by a CaP coating with PLD and solution processes, implants of Group B were subjected to the same CaP coating processes (PLD and solution processes) without FsL irradiation, and implants in Group C were not treated (hereafter referred to as ‘untreated implants’).

### 2.2. FsL Irradiation for Tens-of-Micron- and Submicron-Scale Surface Roughening

**Figure 1:**
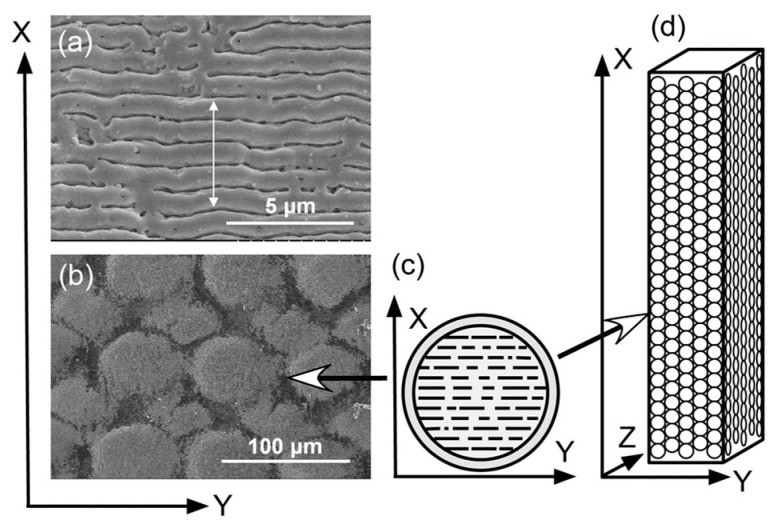
(**a**) High magnification scanning electron microscope (SEM) image of the implant surface, showing linear submicron grooves formed through FsL irradiation. (**b**) Low magnification SEM image of the implant surface. (**c**) Schematic diagram of a crater. (**d**) Schematic diagram of the quadratic prism zirconia implant. The white arrow in (**a**) indicates the length (~4.2 µm) of 5 periods, which means that the length of one period is approximately 840 nm.

The method of FsL irradiation has been described elsewhere [13]. The implants were irradiated through FsL using a Ti:Sapphire chirped-pulse amplification system with a stamp-scan mode at a peak laser fluence *F*_peak_ of approximately 4 J/cm^2^ per pulse. The laser system generated 810 nm centered 80 fs full width at half maximum (FWHM) pulses at a 570 Hz repetition rate. The direction of laser polarization was set parallel to the Y-direction of the implant, as shown in Figure 1. After each spot was irradiated with 40 pulses, the irradiation position was moved by 60 µm in the X-direction. When the laser reached the end of the X-direction, the irradiation position was moved by 30 and 90 µm in the X- and Y-directions, respectively, to irradiate the next row. The 60 µm stepwise irradiation was then restarted in the X-direction, to fully fill the interstices of elliptical craters. The irradiation was repeated until the length of the irradiated area reached 20 mm in the X-direction. In this laser treatment, five samples were placed side by side and the 12.0 mm (Y) × 20 mm (X) plane was irradiated at the same time. All four rectangular planes of the implant were irradiated. After the laser irradiation, the samples were cleaned with cellulose wiper wetted with ethanol, washed ultrasonically three times with acetone, and then air dried.

FsL irradiation generated elliptical craters with a lateral dimension of 90 µm (Y-direction) × 60 µm (X-direction) and a maximum depth of 6.7–7.0 µm, as measured using a confocal laser microscope (VK-9700, Keyence Co., Osaka, Japan) and a scanning electron microscope (SEM; S-4800, Hitachi High-Tech Corporation, Tokyo, Japan). Linear submicron grooves parallel to the Y-direction with a period of around 840 nm (Figure 1a) were formed on the concave surface of the craters (Figure 1b). Approximately half of the surface area exhibited linear submicron grooves, while the other half showed a non-periodic submicron roughness.

### 2.3. Pulsed Laser Deposition of CaP Underlayer

The zirconia implants with and without FsL irradiation (for Groups A and B, respectively) were subjected to PLD for depositing a CaP underlayer using the 4th harmonics of an Nd:YAG laser system (Quanta-Ray Pro-350, Spectra-Physics, Milpitas, CA, USA, wavelength: 266 nm, pulse duration: 10 ns, repetition rate: 10 Hz). A disk of β-tricalcium phosphate (β-TCP: Ca_3_(PO_4_)_2_), 14 mm diameter and 1 mm thick, was used as the target. This experimental configuration for PLD has been described elsewhere [30]. The disk targets were prepared by compacting and sintering β-TCP powders (Olympus Terumo Biomaterials Corp., Tokyo, Japan) at 1100 °C for 1 h in air. The laser beam was focused onto a β-TCP disk target using a planoconvex lens (f = 400 mm) at an incident angle of 45°, and the focused beam size on the target was elliptical with major and minor axis lengths of 270 and 150 µm, respectively. The laser energy was controlled to approximately 19 mJ using a polarizer and a half wave plate, which corresponded to 60 J/cm^2^ with a stability within ±5% on the target surface. The target was moved 60 µm after each shot in the raster scan. Ablated particles were ejected intermittently toward the samples placed 20 mm away from the β-TCP target surface. Four samples placed side by side were treated at the same time at room temperature (20–25 °C), and all four rectangular planes of each sample were coated. Water vapor was introduced into the process chamber to control the stoichiometry of the coating, as discussed below. The water vapor pressure was controlled using a water vapor supply system consisting of a water cell at 20 °C, a capacitance manometer (Type 627A01 (1 Torr full scale), MKS instruments Inc., Andover, MA, USA), and a pressure controller (Type 250, MKS instruments Inc., Andover, MA, USA) with a low flow metering valve, as well as a butterfly valve at the inlet port of the vacuum pump. Depending on the water vapor pressure, the process chamber vacuum was in the range from 1 × 10^–5^ to 0.7 Torr. In a preliminary study using a copper substrate, the Ca/P molar ratio of the CaP coating, measured using an X-ray fluorescence analyzer (SEA5120A, SII NT Co., Chiba, Japan), decreased with increasing water vapor pressure. Based on this preliminary result, the water vapor pressure for the PLD of zirconia implants was set to 0.093 Torr to obtain a CaP underlayer with a Ca/P molar ratio of 1.60–1.67, which is slightly lower than that of stoichiometric hydroxyapatite.

### 2.4. Solution Processing for CaP Growth

After PLD (deposition of the CaP underlayer), the implants for Groups A and B were subjected to the solution process; they were immersed in a supersaturated CaP solution to grow CaP, as described previously [32]. The supersaturated CaP solution was prepared by mixing clinically available infusions and injection fluids using the same method as in [32]: 7% MEYLON^®^ injection (Otsuka Pharmaceutical Co., Ltd., Tokyo, Japan); water for injection (FUSO Pharmaceutical Industries, Ltd., Osaka, Japan); Klinisalz^®^ (KYOWA CritiCare Co., Ltd., Tokyo, Japan); dibasic potassium phosphate injection 20 mEq kit (TERUMO Co., Tokyo, Japan); Ringer’s solution OTSUKA (Otsuka Pharmaceutical Co., Ltd., Tokyo, Japan); calcium chloride corrective injection 1 mEq/mL (Otsuka Pharmaceutical Co., Ltd., Tokyo, Japan); and OTSUKA normal saline (Otsuka Pharmaceutical Co., Ltd., Tokyo, Japan). All samples in Groups A and B were immersed in 200 mL of the solution at 37 °C for 48 h.

### 2.5. Surface Characterization of the Implants

The implant surface was analyzed using a SEM (S-4800, Hitachi High-Tech Corporation, Tokyo, Japan) equipped with an energy-dispersive X-ray analyzer (EDX; EMAX x-act, HORIBA, Ltd., Kyoto, Japan). The calcium and phosphorus contents of the coating were analyzed using an inductively coupled plasma atomic emission spectrometer, after acid dissolution of the coating (ICP: SPS7800, Seiko Instruments, Inc., Chiba, Japan). The thickness of the CaP underlayer (after PLD) and that of the final coating layer (after subsequent solution process) were measured using a laser confocal microscope (VK-X3000, Keyence Co., Osaka, Japan), only for Group B samples. The measurements could not be carried out on Group A samples because of the craters on the sample surface due to FsL irradiation. In addition, since the coating process was the same for both Groups A and B, the thickness of the coating should have been the same for both groups. Prior to the measurements, straight-line portions of the surface layers were scratched using a knife blade, to expose the zirconia surface. The thicknesses of the layers were measured using 3D surface profiles. Five different points per cross-sectional height profile, and five different cross-sectional height profiles were used to calculate average and standard deviation of the thickness of each layer (N = 25).

### 2.6. Implantation in Rabbit Tibia

Eight implants in Groups A, B, and C were randomly implanted in both proximal tibial metaphysis of twelve male Japanese white rabbits (weight range: 2.5–2.8 kg, 14 weeks old) by a single physician who was blinded to the sample identifications. Three implants in each group were used for histomorphometry analysis and five for radiographical analysis, followed by push-out test.

**Figure 2:**
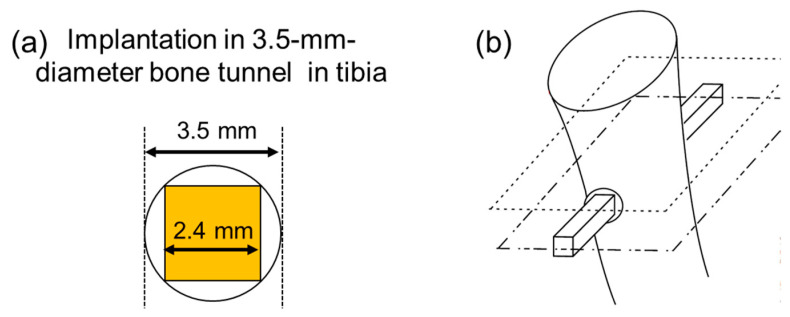
Schematic diagram of implantation of the implant into the proximal tibial metaphysis in a rabbit. (**a**) Front view of the 2.4 mm × 2.4 mm square plane of the implant in the bone tunnel with a diameter of 3.5 mm. (**b**) An overall view of the implant in the proximal tibia.

After an intravenous injection of barbiturate (40 mg/kg body weight), small (10 mm) incisions were aseptically made in the skin at the medial proximal tibia. Bone tunnels 3.5 mm in diameter were then drilled bicortex in both proximal tibial metaphysis. The implants were manually inserted into the tunnels, from the medial to lateral cortex of the tibia (Figure 2). After the implantation, the skin was sutured with a 2-0 nonabsorbable suture. Postoperatively, each animal was allowed free activities in its own cage. All animals were then sacrificed four weeks after the operations.

All the animal experiments and breeding were performed according to conditions approved by the ethics committees of both Ibaraki Prefectural University of Health Sciences and National Institute of Advanced Industrial Science and Technology (AIST), and were in accordance with the National Institutes of Health Guidelines for the Care and Use of Laboratory Animals.

### 2.7. Radiographical Analysis

Radiographs of proximal tibial metaphyses containing the zirconia implants were recorded on imaging plates (Fuji Film, Tokyo, Japan) using a medical Roentgen diagnostics system (DR-150-1, Hitachi Inc., Tokyo, Japan). An X-ray was irradiated perpendicular to the rectangular plane of the implant using a universally rotating sample holder. The bone-to-implant contact ratio was evaluated for the radiographs by a physician blinded to the sample identifications using Image J ver. 1.48. The bone-to-implant contact ratio was defined as the percentage of the length of direct contact between the bone and the implant surface in the total length of the implant within the bone on X-ray radiographs. The length of direct contact was the bone formed on the surface of the implant placed inside the bone and was the sum of the length that was continuous with the bone cortex and in direct contact with the implant. Moreover, bird’s-eye views were prepared from the radiographs by plotting the intensity of X-ray absorption at each X‒Y point on the *Z*-axis.

### 2.8. Push−Out Tests

After radiographical analysis, the failure load of the bone–implant interface was measured in push-out tests using a universal testing machine (Type EZ-L, Shimazu Co., Kyoto, Japan). The proximal tibial metaphyses containing zirconia implants were fixed on a tilting stage. A compressive load was applied to the implant at a cross-head speed of 2 mm/min in the direction of its *X*-axis with the use of a square socket with a steel ball. Failure strength was calculated by dividing the failure load (Fmax) by the area of the implant surface within the bone tunnel (S). The surface area was estimated by measuring the four lengths (in the X-direction) of the implant within the bone tunnel using a caliper and multiplying the sum (sum of the four lengths) by 2.4 mm.

### 2.9. Alizarin Red Staining of the Zirconia Implant

After measuring the failure load, the zirconia implants were carefully removed from the tibial metaphyses by cutting the bone just above the proximal side of the implants. The removed implants were immersed in 10% phosphate-buffered formalin solution for 10 days to fix the tissue, rinsed twice with calcium- and magnesium-free phosphate-buffered saline (PBS (‒)), immersed in a 1% alizarin red solution at pH 6.35 for staining calcium ions with alizarin red, and finally rinsed with PBS (‒). Thickness of the alizarin red stained area was measured using a laser confocal microscope (OLS-4100, Olympus, Tokyo, Japan). Images of the stained implants were captured using a stereoscopic microscope (SZX16, Olympus Co., Japan), and analyzed for stained area using Image J ver. 1.48.

### 2.10. Histomorphometric Analysis through SEM

The proximal tibial metaphyses containing zirconia implants were fixed in 10% phosphate-buffered formalin solution, dehydrated in serial concentrations of ethanol (70, 80, 90, and 99.5 vol%) and acetone, and then embedded in an acrylic resin (poly(methyl methacrylate)) through polymerization. The cured resin specimens were cut parallel to the X‒Z plane of the implant and perpendicular to the bone axis using a micro-cutting machine (BS-300CL, EXAKT Advanced Technologies, Norderstedt, Germany). The cutting blade was inserted at a position slightly away from the center line of the Y‒Z plane considering the cutting allowance (~0.4 mm), so that the longitudinal rectangular section at the center of the implant (~1.2 mm distant from the X‒Z plane) was exposed. The surfaces of the resin specimens including the half-cut implants were polished to mirror quality using a micro-grinding machine (MG-400 CS, EXAKT Advanced Technologies, Norderstedt, Germany) and #4000 polishing paper. The interfaces of the bone and implant in 2 mm regions (four regions per resin specimen) from the outer edges of the cortical bone were observed using SEM in backscattered electron imaging mode, and the captured images were analyzed using Image J. The bone-to-implant contact ratio was calculated as the percentage of the length of the bone-contacting implant surface of the total length of the implant surface in the test region. The bone-contacting implant surface was determined as the implant surface where the gap with the bone was ≤5 μm.

### 2.11. Statistical Analysis

All the data from each group were analyzed using generalized linear models. Statistical analysis was performed using IBM SPSS 28.0, with a 5% level of statistical significance.

## 3. Results

### 3.1. Surface Characterization of the Implants

**Figure 3:**
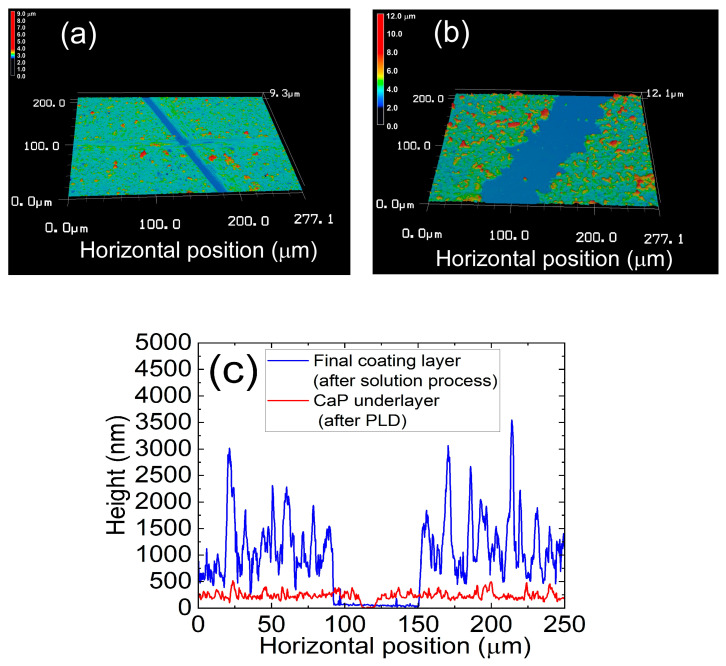
Three-dimensional profiles (from a laser confocal microscope) of the partially scratched implant (Group B) surfaces after (**a**) PLD and (**b**) subsequent solution processing in false color, and (**c**) representative cross-sectional height profiles of the CaP underlayer (after PLD) and those of the final coating layer (after subsequent solution process) including the scratched portions.

The implants in Groups A and B were fully coated with CaP layers with similar surface morphology and chemical composition. Figure 3 shows 3D profiles (from a laser confocal microscope) of the partially scratched implant (Group B) surfaces after (a) PLD and (b) subsequent solution processing in false color. The diagonal straight lines with blue color are the scratched portions with exposed zirconia surface on the implants. Representative cross-sectional height profiles of the CaP under-layer (after PLD) and final coating layer (after subsequent solution process) including the scratched portions are shown in Figure 3c. Micron-scale protrusions observed on the final coating layer correspond to the particles (red dots) on its 3D surface profile (Figure 3c). According to the quantitative analysis, the CaP underlayer on the implant deposited through PLD was 204 ± 22 nm thick, which increased to 992 ± 195 nm after the subsequent solution process.

**Figure 4:**
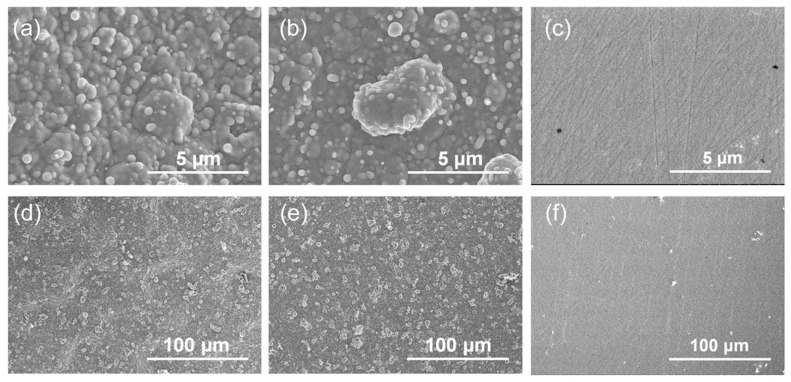
SEM images, with (**a**–**c**) high and (**d**–**f**) low magnifications, of the surfaces of the (**a**,**d**) CaP-coated implant with FsL irradiation (Group A), (**b**,**e**) CaP-coated implant without FsL irradiation (Group B), and (**c**,**f**) untreated implant (Group C).

The resulting implants in both Groups A and B showed micro-rough surfaces (Figure 4a,b,d,e), whereas those in Group C showed a flat surface (Figure 4c,f). On the implants in Group A, the linear submicron grooves formed by FsL irradiation (Figure 1a) were invisible due to the overlaid CaP layer, although the elliptical crater-like structures were barely recognizable, as shown in Figure 4d. The CaP-coated crater had a size of about 90 × 60 µm in diameter and 5 µm in depth.

**Figure 5:**
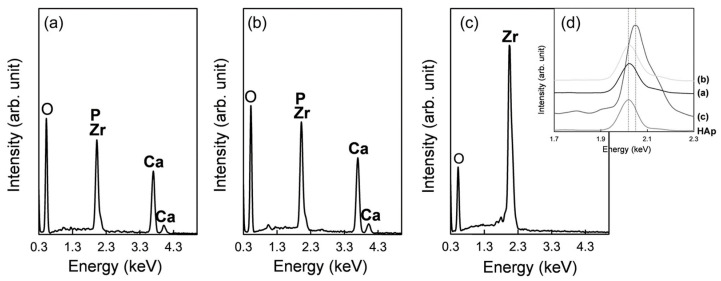
EDX spectra of the surfaces of the (**a**) CaP-coated implant with FsL irradiation (Group A), (**b**) CaP-coated implant without FsL irradiation (Group B), and (**c**) untreated implant (Group C). (**d**) Magnified EDX spectra of (**a**–**c**) and of sintered hydroxyapatite (HAp) in the range 1.7–2.3 keV.

EDX analysis revealed that the surface layers on the Group A and B implants were composed of CaP (Figure 5). Detailed analysis of the peak positions of P and Zr in the range 1.7–2.3 keV confirmed the presence of P on the surfaces of these implants (Figure 5d). No noticeable differences in chemical composition and morphology of the CaP layers were found between Groups A and B (Figure 5a,b); the critical difference was the presence (Group A) or absence (Group B) of surface waviness, which reflected the tens-of-micron-sized craters formed by FsL irradiation. The quantitative analysis of Ca and P using ICP showed Ca and P contents of 132.4 µg and 58.1 µg, respectively, with a Ca/P molar ratio of 1.76 for Group A, and 171.4 µg and 73.3 µg, respectively, with a Ca/P molar ratio of 1.81 for Group B.

### 3.2. Radiographical Analysis

**Figure 6:**
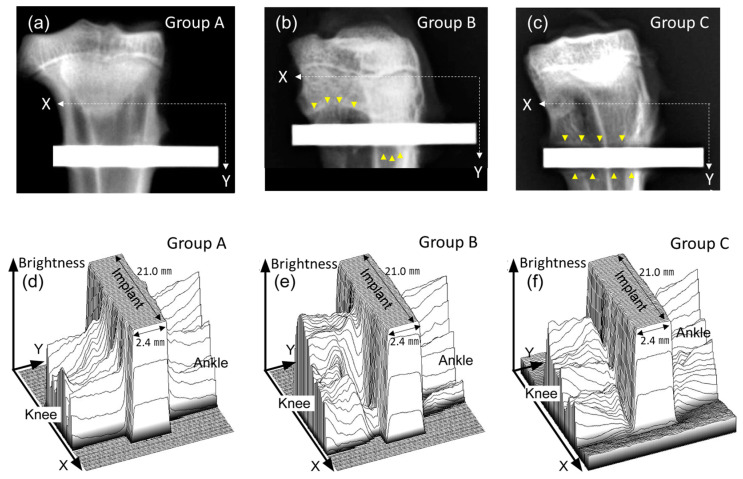
X-ray radiographs of the implants in Groups A (**a**), B (**b**), and C (**c**) in the tibias, and their bird’s-eye views, where the brightness (related to the intensity of X-ray absorption) of each X‒Y plane in (**a**–**c**) was converted into height in (**d**–**f**), respectively. The yellow arrows are part of the radiolucent line.

Typical radiographs of the proximal tibial metaphyses containing the implants in Groups A, B, and C are shown in Figure 6a–c, respectively. In the radiographs of Groups B and C, significant radiolucent lines were observed around the implant, whereas no clear radiolucent line was found around the implants in Group A. Radiolucent lines are often associated with intervening fibrous tissue formation between an implant and bone. The bird’s-eye views in Figure 6d–f visualize the relative intensity of X-ray absorption in Figure 6a–c, respectively. The views of Groups B and C show valleys of low intensity in the close vicinity of the implants (corresponding to the radiolucent lines in Figure 6b,c) as shown in Figure 6e,f; on the other hand, no such valley was apparent in the vicinity of the Group A implants (Figure 6d). These results suggest that the Group A implants showed better bone-to-implant contact in the rabbit tibias than the implants in Groups B and C, which was confirmed through quantitative radiographical analysis, as described below.

**Figure 7:**
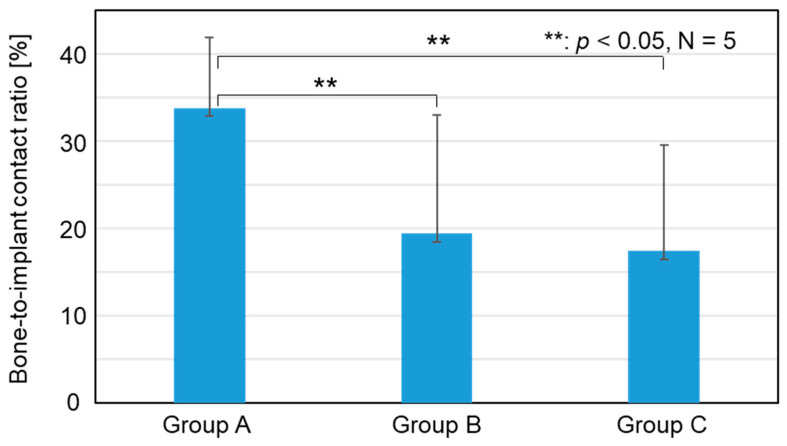
Bone-to-implant contact ratio of Groups A, B, and C measured through radiographical analysis.

According to the radiographical analysis, the bone-to-implant contact ratios were 33.8 ± 8.0, 19.4 ± 13.5, and 17.4 ± 12.0% for Groups A, B, and C, respectively, as shown in Figure 7. The bone-to-implant contact ratio for Group A was significantly higher than those for Groups B (*p* = 0.027) and C (*p* = 0.012). No significant difference was found between Groups B and C (*p* = 0.754).

### 3.3. Push−Out Tests

**Figure 8:**
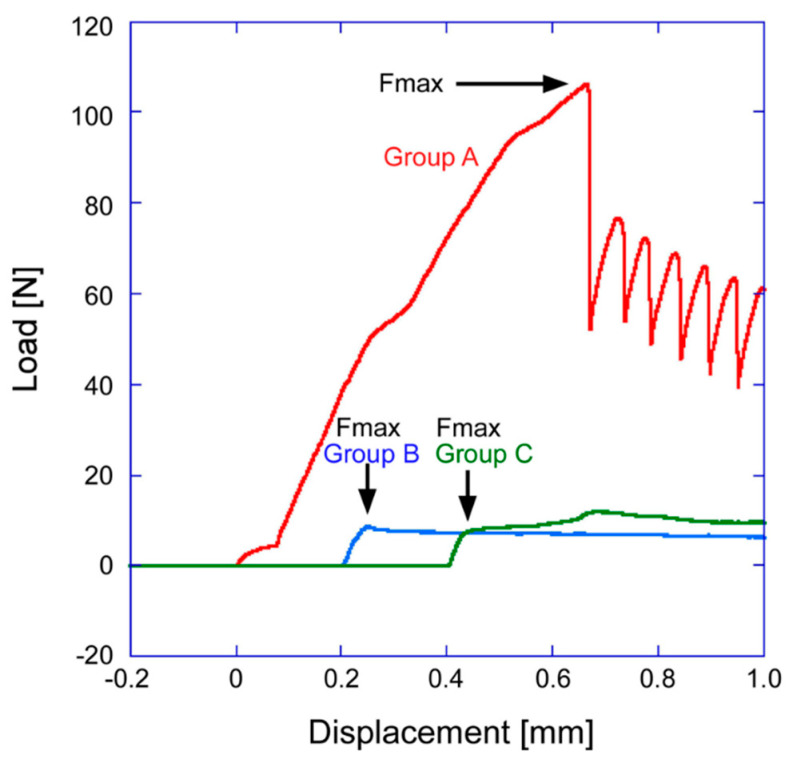
Typical load−displacement curves in the push−out tests for Groups A, B, and C.

Typical load−displacement curves are shown in Figure 8. A sawtooth pattern invariably only appeared on the curve for Group A. The distance between the two neighboring sawtooth peaks was approximately 56 µm, which is almost the same as the distance of 60 µm between the centers of the elliptical craters generated through FsL irradiation.

**Figure 9:**
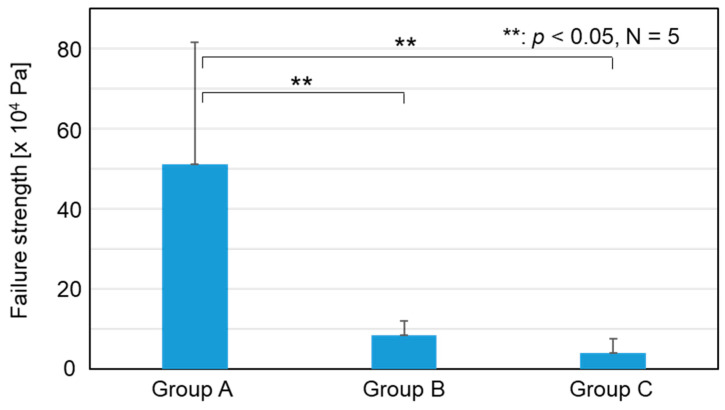
Failure strength of the implants in Groups A, B, and C in the push−out tests.

The failure strength (× 10^4^ Pa) in the push-out test was 51.1 ± 30.6, 8.3 ± 3.7, and 3.9 ± 3.6 for Groups A, B, and C, respectively. The failure strength for Group A was significantly higher than that for Groups B (*p* < 0.001) and C (*p* < 0.001), as shown in Figure 9. No significant difference was found between Groups B and C (*p* = 0.667, Figure 9). These results indicate that the Group A implants showed higher adhesion strength with the bone tissue compared to the implants in Groups B and C.

### 3.4. Alizarin Red Staining of the Implants after Push-Out Tests

**Figure 10:**
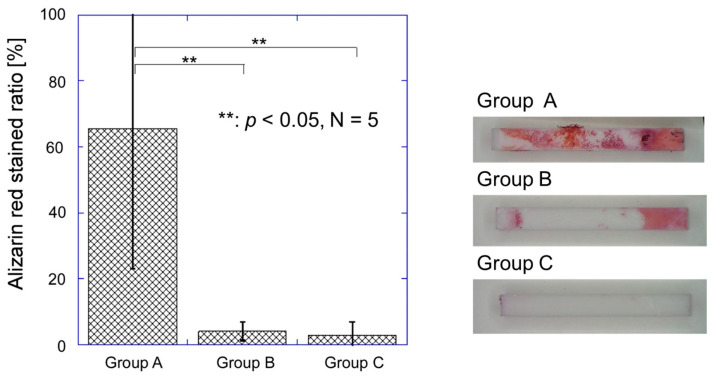
Ratio of the alizarin red stained area to the whole surface area of the implants in Groups A, B, and C after push−out tests (**left**). Typical macroscopic image of alizarin red stained implants in Groups A, B, and C after push-out tests (**right**).

After the push−out tests, the Group A implants removed from the tibial metaphyses were more widely and strongly stained with alizarin red (calcium indicator) than those of Groups B and C (Figure 10). The implants in Group A had a strongly stained region on a large part of the surface (Figure 10 right). The stained portion on the implants in Group A had a thickness from 5 to 10 µm, which was thicker than the estimated initial thickness of the CaP coating layer (approximately 1 µm). Thus, the main component of this stained portion was likely fractured bone tissue. In contrast, the implants in Groups B and C were hardly stained with alizarin red (Figure 10 right), suggesting that the amount of bone tissue on their surfaces was relatively small. The ratio of stained area for Group A was significantly larger than that for Groups B (*p* < 0.001) and C (*p* < 0.001) (Figure 10 left). No significant difference was found between Groups B and C in the ratio of the stained area (*p* = 0.144, Figure 10 left).

### 3.5. Histomorphometric Analysis through SEM

**Figure 11:**
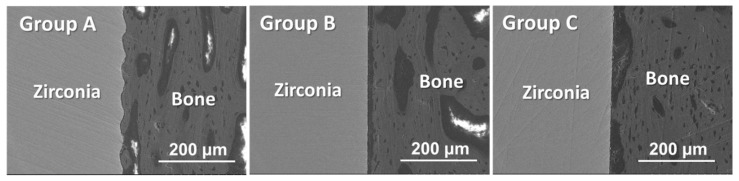
SEM images (backscattered electron images) of the histological sections showing the bone–implant interface of Groups A, B, and C.

Histomorphometric analysis through SEM revealed better contact of the cortical bone with the implant in Group A than for the implants in Groups B and C. Figure 11 shows typical SEM images of the histological sections, showing the interfaces between the cortical bone and zirconia implants. The bone tissue directly contacted the implant surface in a wider region for Group A than for Groups B and C. In Group A, bone tissue was even observed in the elliptical craters on the implant surface created through FsL irradiation. An electron-sparse layer was seen between the zirconia and bone in Groups B and C. As shown in Figure S1, the electron-sparse layer was rich in carbon and contained no calcium. Thus, the electron-sparse layer was that of an intervening soft tissue. Such an electron-sparse layer was scarcely seen between the zirconia and bone in Group A.

**Figure 12:**
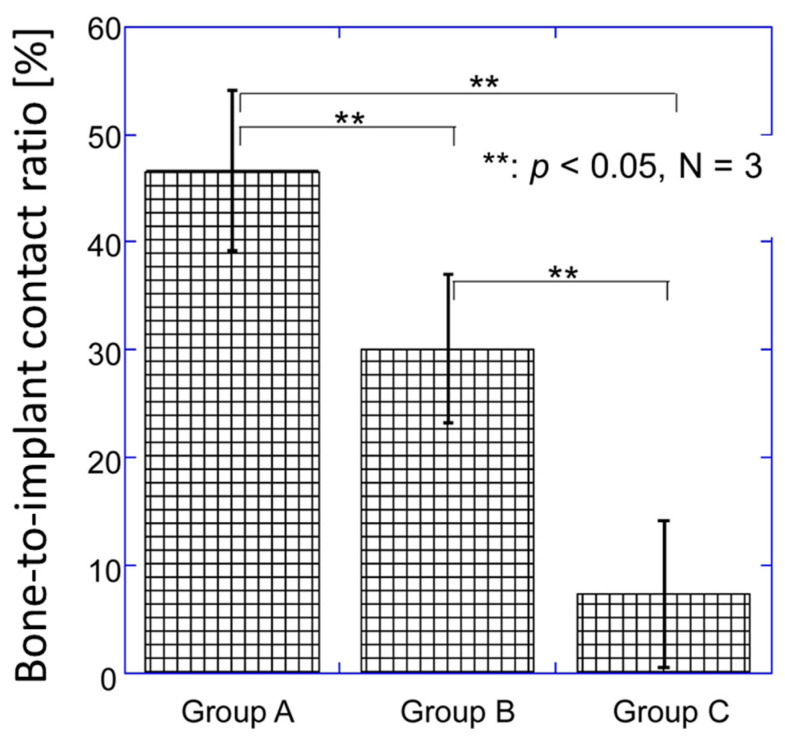
Bone-to-implant contact ratio of Groups A, B, and C.

According to the SEM and EDX analyses, residual CaP layers were not clearly identified on the implants for either Group A or B. As shown in Figure 12, the bone-to-implant contact ratio of Group A was significantly higher than that of Groups B (*p* < 0.001) and C (*p* < 0.001). The bone-to-implant contact ratio of Group B was also significantly higher than that of Group C (*p* < 0.001).

## 4. Discussion

FsL irradiation followed by CaP coating on zirconia improved the bone-bonding ability of zirconia. In Group A, FsL irradiation followed by CaP coating generated a micro-ruggedness consisting of partially-overlapped elliptical craters and an approximately 1 µm-thick CaP coating layer on the zirconia. The same CaP coating thickness was deposited on the Group B implants without FsL irradiation. The four different in vivo analyses conducted in the present study (radiographical analysis, push−out test, alizarin red staining, and histomorphometric analysis) demonstrated a much improved bone-bonding ability of zirconia in Group A over that in Groups B and C. In the histomorphometric analysis through SEM (Figure 11), the bone-to-implant contact ratio was higher in Group B than in Group C, most likely because of the CaP coating layer. However, no significant differences between Groups B and C were observed in the radiographical analysis, push-out test, and alizarin red staining. Therefore, FsL irradiation played a critical role in enhancing the bone-bonding ability of CaP-coated zirconia.

FsL irradiation generated a surface architecture that enhanced the osteogenic differentiation of mesenchymal stem cells (MSCs). Studies have shown that surface architectures and roughness similar to that of an osteoclast resorption pit are sensed by MSCs in contact with proteins adsorbed on an implant surface, causing MSCs to undergo osteogenic differentiation [28]. An osteoclast resorption pit has an area of approximately 700–7800 μm^2^, lateral diameter of 30–100 μm, and depth of ~10 μm [33]. Elliptical craters formed through FsL irradiation on zirconia under conditions similar to those of the present study had an area of approximately 5000 μm^2^, lateral diameter of ~80 μm, and depth of ~10 μm, enhancing the osteogenic differentiation of MSCs [28]. In the present study, the CaP-coated craters on zirconia had an area of approximately 4200 μm^2^, lateral diameter of 90 × 60 μm, and depth of ~5 μm.

The surface architecture generated through FsL irradiation supported the osteoconduction of the CaP layer, which resulted in an improvement in the osteointegration of the zirconia in the present animal model. Osteointegration is defined as the formation of direct contact between the implant surface and bone tissue without intervening fibrous tissue, being a key factor for the long-term success of the implant [34]. In the animal experiment, a quadratic prism implant was inserted into a cylindrical bone tunnel (see Figure 2). Thus, the implant was not fixed tightly in the bone tunnel at the time of implantation; the gap between the implant and the inner wall of the bone tunnel was from 0.05 mm to 0.55 mm. Even under such loose-contact conditions, the zirconia in Group A showed significantly higher failure strengths than those in Groups B and C in the push-out test 4 weeks after implantation. This was because the zirconia in Group A was integrated more firmly into the newly formed bone. Although it is unclear whether the CaP layer remained unresorbed and whether osteoconduction was involved four-weeks after implantation, enhanced osteointegration was achieved on the zirconia in Group A. In contrast, the zirconia in Groups B and C exhibited limited osteoconduction or osteointegration, as evidenced by the radiolucent line around the implant in the radiographical analysis, and the lower bone-to-implant contact ratios and electron-sparse layer in the histomorphometric analysis. A radiolucent line and the electron-sparse layer are often associated with intervening fibrous tissue-formation, which means no osteoconduction occurred where the fibrous tissue was formed.

**Figure 13:**
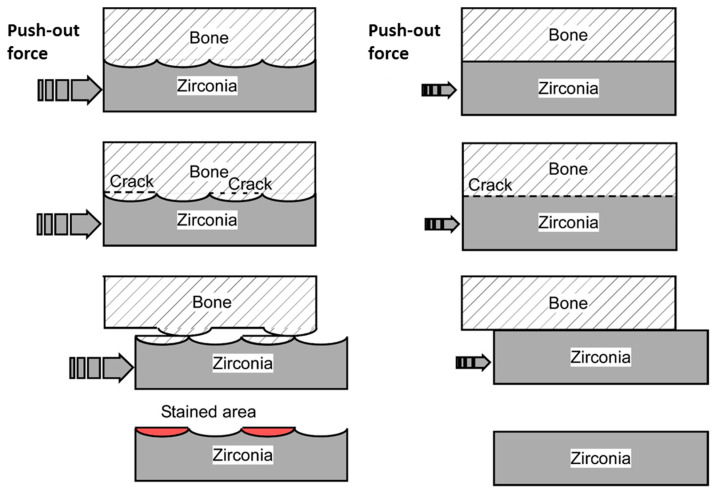
Putative schematic diagrams of failure mode in the push−out tests (upper three rows) and subsequent alizarin red staining (bottom row) for Group A (left column) and Groups B and C (right column).

FsL irradiation of zirconia also improved the mechanical interlocking, to enhance the adhesion strength between the zirconia and the CaP layer, as well as between the zirconia and bone. FsL irradiation of zirconia not only induced tens-of-micron-sized craters and submicron grooves but also surface wetting via ablation plasma, enabling the zirconia to completely and strongly bond to the CaP layer [13]. The strong adhesion may also be attributed to the mechanical interlocking effects due to the surface architecture generated by FsL irradiation [13]. Moreover, new bone was formed in the elliptical craters in Group A (Figure 11). The resulting mechanical interlocking structure contributed to the improved adhesion strength between the zirconia and bone, as evidenced by the higher failure strength in the push-out test. Putative schematic diagrams of the failure mode in the push-out tests for Groups A, B, and C are illustrated in Figure 13. In Group A, cracks were generated both in the newly formed bone and at the bone–implant interface. The resulting protruding bone and the craters on the implant caused the sawtooth pattern in the load–displacement curve after fracture, due to improved mechanical interlocking (Figure 8). However, in Groups B and C, the cracks were mostly generated at the bone–implant interface, as shown in the righthand diagram of Figure 13. In the subsequent alizarin red staining, the zirconia in Group A had a larger stained area than those in Groups B and C. Furthermore, the stained portion of zirconia in Group A was 5–10-times thicker than the CaP layer coated on the zirconia. Therefore, the stained portion in Group A mainly consisted of bone tissue detached from the host bone because of fractures within the host bone. The bone-to-zirconia adhesion strength in Group A was as high as that inducing host bone fracture in the push-out test. This enhanced bone-to-zirconia adhesion strength was partly due to the improved mechanical interlocking caused by the surface architecture generated through FsL irradiation.

Clinically, a zirconia knee prosthesis requires cementation for fixation with the bone tissue. However, fixation with bone cements involves the risk of complications (bone cement implantation syndrome) such as hypoxia, hypotension, consciousness disturbance, and death in the worst case [11]. The incidence of bone cement implantation syndrome is 24% in total hip arthroplasty (THA) and 28% in total knee arthroplasty (TKA) [11]. Various CaP coating techniques have been proposed to improve the bone-bonding ability of zirconia-based implants, thereby realizing cement-less zirconia joints [12,13,14,15,16,17,18,19,20,21]. Compared to previous techniques, the present surface modification technique utilizing FsL irradiation followed by CaP coating has the advantages of strong coating adhesion [13], supporting osteogenic cell-differentiation [28], and a mechanical interlocking effect, ensuring firm osteointegration without using any toxic reagents such as hydrofluoric acid [12]. The disadvantage of FsL irradiation is the negative impact on the mechanical properties of zirconia [35]. However, this disadvantage can be overcome by improving the manufacturing process of zirconia, reaching mechanical and crystallographic properties that meet the ISO 13556:2015 standard after FsL irradiation (Tables S1 and S2, and Figures S2–S5). As verified in the push-out tests (Figure 9 and Figure 10), the Group A implants showed strong adhesion with the bone tissue, even without cementation. Strong bone-to-implant adhesion is essential for reducing the risk of loosening and ensuring long-term survival. Taken together, application of the present surface modification technique to the bone-contacting region of zirconia-based implants might successfully lead to the development of cement-less zirconia-based joints and zirconia dental implants.

This study had several limitations. Animal experiments were carried out on a small number of rabbits. In the future, the number of animals must be increased, and the experiments must be conducted on larger animals prior to clinical trials. Moreover, this study was limited to a short follow-up period; long-term evaluation is necessary in the future. Third, implants with FsL irradiation and without CaP coating were not considered. In this study, CaP-coated zirconia with and without FsL irradiation was tested to verify the effect of FsL irradiation. The effect of FsL irradiation alone could be a topic for future study.

## 5. Conclusions

FsL irradiation was carried out on zirconia to produce micro-/submicron surface architectures, followed by CaP coating using PLD and solution processes. The FsL-irradiated and CaP-coated zirconia (Group A) showed improved osteointegration and stronger adhesion with the bone tissue in rabbits than the untreated zirconia (Group C) and CaP-coated zirconia without FsL irradiation (Group B). The bone-to-zirconia adhesion in Group A was so strong that host bone fracture occurred in the push-out test. The present surface modification technique could be useful for developing cement-less zirconia-based joints and zirconia dental implants.

## Data Availability

The datasets generated and/or analyzed during the current study are available from the corresponding author upon reasonable request.

## Supplementary Materials

The following supporting information can be downloaded at: https://www.mdpi.com/article/10.3390/jfb15020042/s1, Figure S1: SEM image and EDX line scan profiles (carbon and calcium signals) of the histological section for the bone-implant interface of Group B; Figure S2: Four-point bending strength for zirconia compacted using the additional HIP technique with and without subsequent FsL irradiation, and using the cold isostatic pressing (CIP) technique with and without subsequent FsL irradiation. The compacted temperature was set at 1350 °C for CIP in ambient air and at 1300 °C for the additional HIP treatment in a 147-MPa Ar atmosphere. The four-point bending strength was measured in accordance with ISO 14704 standard (left). FsL irradiation was made on one of the rectangular surfaces. The FsL-irradiated surface was set on the tension side in the bending test (left). Four-point bending strength of zirconia compacted using the additional HIP technique with subsequent FsL irradiation meets the ISO 13556:2015 standard requirement for zirconia implants (right). Reprinted and slightly modified from [36] Copyright 2017, and [37] Copyright 2019 with permission from Japan Laser Processing Society; Figure S3: Four-point bending strength for zirconia compacted using the additional HIP technique and subjected to accelerated aging in hot water (HIP+HW) and that compacted using HIP and subjected to FsL irradiation and accelerated aging in hot water (HIP+FsL+HW). Set-up of specimens and four-point bending test were the same as those in Figure S2. The accelerated aging in hot water was performed in accordance with ISO 13556:2015 standard. After accelerated aging, four-point bending strength of zirconia compacted using the additional HIP technique and subjected to FsL irradiation meets the requirement of ISO 13556:2015 standard for zirconia implants; Figure S4: X-ray diffraction (XRD) patterns for zirconia compacted using the additional HIP technique (HIP), and that subsequently subjected to only FsL irradiation (HIP+FsL) or both FsL irradiation and accelerated aging in hot water (HIP+FsL+HW). The labels “t” and “m” indicate the tetragonal and monoclinic crystal phases, respectively. The monoclinic and tetragonal phases were identified based on the data from The International Centre for diffraction data (ICDD) 01-070-8379 (Baddeleyite, syn) and ICDD 01-081-1544 (Zirconium Oxide), respectively; Figure S5: Monoclinic crystal phase ratio Rm for zirconia compacted using the additional HIP technique (HIP), and that subsequently subjected to only Fs irradiation (HIP+FsL) or Fs irradiation plus accelerated aging in HW (HIP+FsL+HW). Each Rm was calculated from the XRD peak areas for monoclinic and tetragonal phases using ref. [38]. The peak areas for monoclinic and tetragonal phases were analyzed using XRD analysis software (PDXL, Rigaku Co., Tokyo, Japan) with ICDD 01-070-8379 (Baddeleyite, syn) and ICDD 01-081-1544 (Zirconium Oxide), respectively. The Rm data for HIP+FsL were reproduced from [36]. The Rm values meet the ISO 13556:2015 standard requirement for zirconia implants; Table S1: Weibull parameters of four-point bending strength in Figure S2 for zirconia compacted using the additional hot isostatic pressing (HIP), and those subsequently subjected to FsL irradiation (HIP+FsL). Weibull parameters for “HIP+FsL” meet the requirements of ISO 13556:2015 standard for zirconia implants; Table S2: Number of fractured specimens after cyclic fatigue test in accordance with ISO 13556:2015 standard for zirconia implants reproduced from [37].
