# Supplementary material for: Femtosecond Laser Irradiation to Zirconia Prior to Calcium Phosphate Coating Enhances Osteointegration of Zirconia in Rabbits
Source: J Funct Biomater. 2024 Feb 11;15(2):42. doi: 10.3390/jfb15020042 (PMC10889465; doi:10.3390/jfb15020042)
Supplement: Supplementary file 1 [file jfb-15-00042-s001.zip › jfb-2802877-supplementary.pdf]

Supplementary materials

Femtosecond Laser Irradiation to Zirconia Prior to Calcium Phosphate Coating Enhances Osteointegration of Zirconia in Rabbits

Hirotaka Mutsuzaki [1,2,*], Hidehiko Yashiro [3], Masayuki Kakehata [3], Ayako Oyane [4] and Atsuo Ito [5]

1 Center for Medical Science, Ibaraki Prefectural University of Health Sciences, 4669-2 Ami, Ibaraki 300-0394, Japan
2 Department of Orthopaedic Surgery, Ibaraki Prefectural University of Health Sciences Hospital, 4773 Ami, Ibaraki 300-0331, Japan
3 Research Institute for Advanced Electronics and Photonics, National Institute of Advanced Industrial Science and Technology (AIST), AIST Tsukuba Central 2, 1-1-1 Umezono, Tsukuba, Ibaraki 305-8568, Japan; hidehiko.yashiro@aist.go.jp (H.Y.); kakehata-masayuki@aist.go.jp (M.K.)
4 Nanomaterials Research Institute, National Institute of Advanced Industrial Science and Technology (AIST), AIST Tsukuba Central 5, 1-1-1 Higashi, Tsukuba, Ibaraki 305-8565, Japan; a-oyane@aist.go.jp
5 Health and Medical Research Institute, National Institute of Advanced Industrial Science and Technology (AIST), AIST Tsukuba Central 6, 1-1-1 Higashi, Tsukuba, Ibaraki 305-8566, Japan; atsuo-ito@aist.go.jp
* Correspondence: mutsuzaki@ipu.ac.jp; Tel.: +81-29-888-4000; Fax: +81-29-840-2301

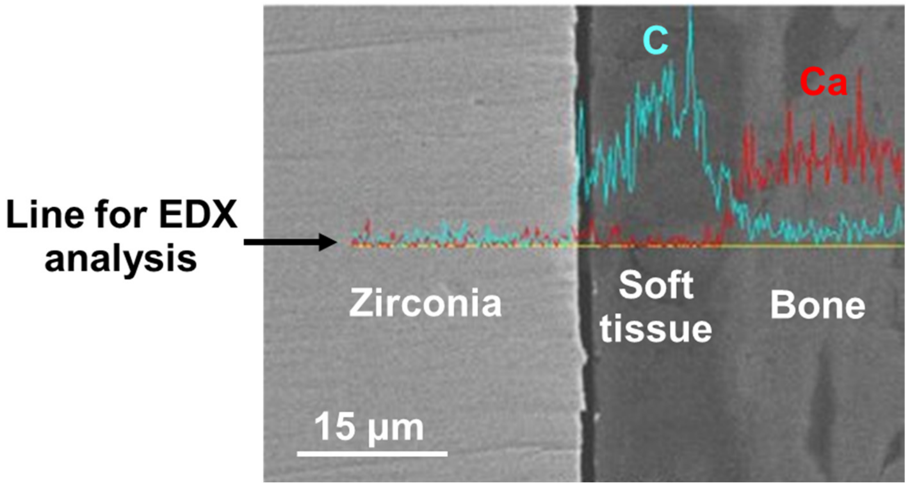

Figure S1. SEM image and EDX line scan profiles (carbon and calcium signals) of the histological section for the bone-implant interface of Group B.

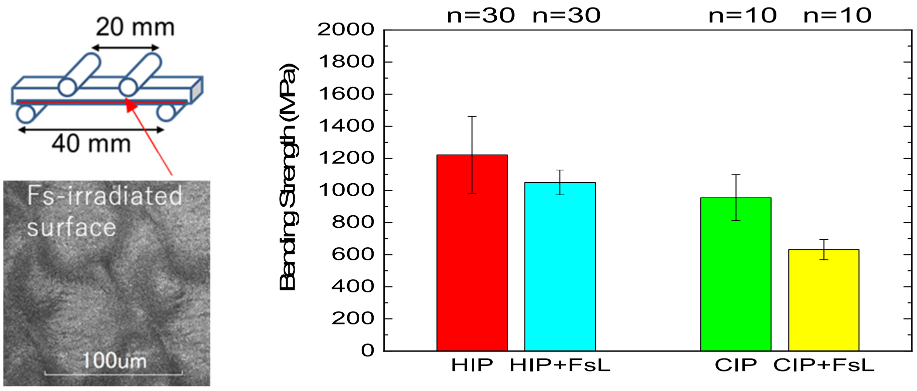

Figure S2. Four-point bending strength for zirconia compacted using the additional HIP technique with and without subsequent FsL irradiation, and using the cold isostatic pressing (CIP) technique with and without subsequent FsL irradiation. The compacted temperature was set at 1350 ºC for CIP in ambient air and at 1300 ºC for the additional HIP treatment in a 147-MPa Ar atmosphere. The four-point bending strength was measured in accordance with ISO 14704 standard (left). FsL irradiation was made on one of the rectangular surfaces. The FsL-irradiated surface was set on the tension side in the bending test (left). Four-point bending strength of zirconia compacted using the additional HIP technique with subsequent FsL irradiation meets the ISO 13556:2015 standard requirement for zirconia implants (right). Reprinted and slightly modified from [36] Copyright 2017, and [37] Copyright 2019 with permission from Japan Laser Processing Society.

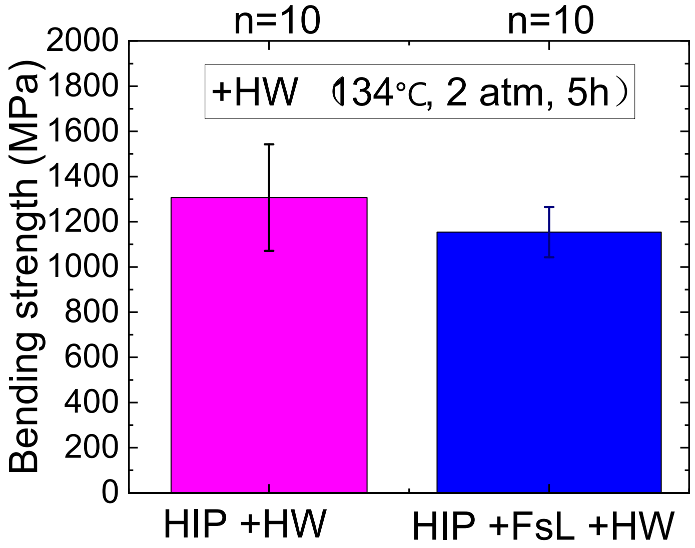

Figure S3. Four-point bending strength for zirconia compacted using the additional HIP technique and subjected to accelerated aging in hot water (HIP+HW) and that compacted using HIP and subjected to FsL irradiation and accelerated aging in hot water (HIP+FsL+HW). Set-up of specimens and four-point bending test were the same as those in Fig. S2. The accelerated aging in hot water was performed in accordance with ISO 13556:2015 standard. After accelerated aging, four-point bending strength of zirconia compacted using the additional HIP technique and subjected to FsL irradiation meets the requirement of ISO 13556:2015 standard for zirconia implants.

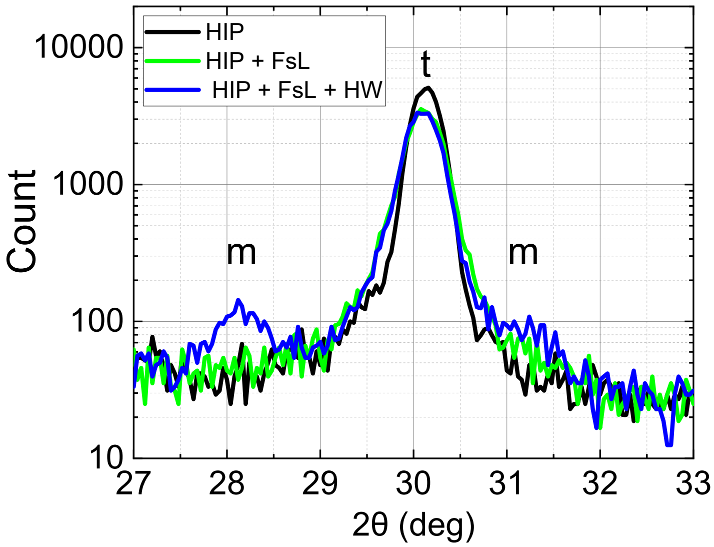

Figure S4. X-ray diffraction (XRD) patterns for zirconia compacted using the additional HIP technique (HIP), and that subsequently subjected to only FsL irradiation (HIP+FsL) or both FsL irradiation and accelerated aging in hot water (HIP+FsL+HW). The labels "t" and "m" indicate the tetragonal and monoclinic crystal phases, respectively. The monoclinic and tetragonal phases were identified based on the data from The International Centre for diffraction data (ICDD) 01-070-8379 (Baddeleyite, syn) and ICDD 01-081-1544 (Zirconium Oxide), respectively.

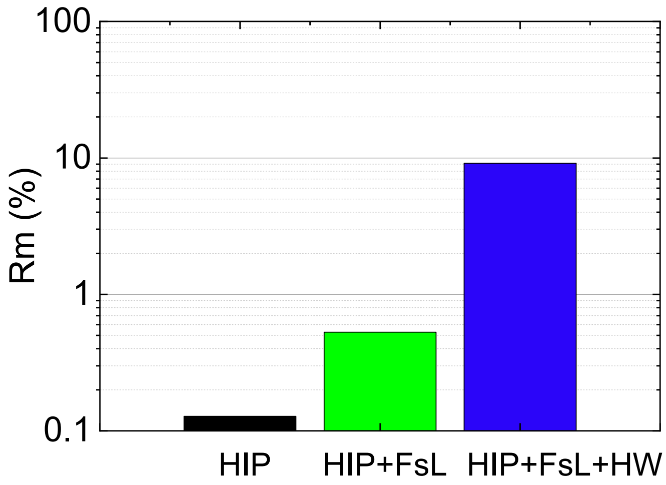

Figure S5. Monoclinic crystal phase ratio Rm for zirconia compacted using the additional HIP technique (HIP), and that subsequently subjected to only Fs irradiation (HIP+FsL) or Fs irradiation plus accelerated aging in HW (HIP+FsL+HW). Each Rm was calculated from the XRD peak areas for monoclinic and tetragonal phases using ref. [38]. The peak areas for monoclinic and tetragonal phases were analyzed using XRD analysis software (PDXL, Rigaku Co., Tokyo, Japan) with ICDD 01-070-8379 (Baddeleyite, syn) and ICDD 01-081-1544 (Zirconium Oxide), respectively. The Rm data for HIP+FsL were reproduced from [36]. The Rm values meet the ISO 13556:2015 standard requirement for zirconia implants.

Table S1. Weibull parameters of four-point bending strength in Fig. S2 for zirconia compacted using the additional hot isostatic pressing (HIP), and those subsequently subjected to FsL irradiation (HIP+FsL). Weibull parameters for "HIP+FsL" meet the requirements of ISO 13556:2015 standard for zirconia implants.

	HIP	HIP+FsL
Weibull modulus, m	6.2	14.1
Weibull scale parameter (MPa)	1316	1085
Number of specimens	30	30

Table S2. Number of fractured specimens after cyclic fatigue test in accordance with ISO 13556:2015 standard for zirconia implants reproduced from [37].

	HIP	HIP+FsL
Number of tested specimens	5	5
Number of fractured specimens	0	0

References

[36] akehata, M.; Oyane, A.; Yashiro, H.; Ito, A.; Okazaki, Y.; Torizuka, K. Bending strength and cyclic fatigue tests of yttria-stabilized zirconia ceramics modified with femtosecond-laser induced periodic surface structures for medical implants. In Proceedings of the 8th International Congress on Laser Advanced Material Processing (LAMP2019), Hiroshima, Japan, 21–24 May 2019; 2019, #19-063. This paper is available upon request to Japan Laser Processing Society. Available online: http://www.jlps.gr.jp/symposium/information/index.html (accessed on 3 September 2019).

[37] Kakehata, M.; Ito, A.; Yashiro, H.; Oyane, A.; Torizuka, K. Effect of femtosecond laser surface treatment on bending strength of yttria-stabilized zirconia ceramics. In Proceedings of the 18th International Symposium on Laser Precision Microfabrication (LPM2017), Toyama, Japan, 5–8 June 2017; 2017, #17-76. This paper is available upon request to Japan Laser Processing Society. Available online: http://www.jlps.gr.jp/symposium/information/index.html (accessed on 28 July 2017). In the proceedings, correct parameters as b = 4 mm, d = 3 mm in Equation(1).

[38] Toraya, H.; Yoshimura, M.; Somiya, S. Calibration curve for quantitative analysis of the monoclinic-tetragonal ZrO_2 system by X-ray diffraction. *J Am Ceram Soc* **1984**, *67*, C119.
